# Design and pre-clinical profiling of a *Plasmodium falciparum *MSP-3 derived component for a multi-valent virosomal malaria vaccine

**DOI:** 10.1186/1475-2875-8-314

**Published:** 2009-12-30

**Authors:** Marco Tamborrini, Markus S Mueller, Sabine A Stoffel, Nicole Westerfeld, Denise Vogel, Francesca Boato, Rinaldo Zurbriggen, John A Robinson, Gerd Pluschke

**Affiliations:** 1 Swiss Tropical Institute, Molecular Immunology, CH-4002 Basel, Switzerland; 2 Pevion Biotech Ltd., CH-3036 Ittigen/Bern, Switzerland; 3 Institute of Organic Chemistry, University of Zurich, CH-8057 Zurich, Switzerland

## Abstract

**Background:**

Clinical profiling of two components for a synthetic peptide-based virosomal malaria vaccine has yielded promising results, encouraging the search for additional components for inclusion in a final multi-valent vaccine formulation. This report describes the immunological characterization of linear and cyclized synthetic peptides comprising amino acids 211-237 of *Plasmodium falciparum *merozoite surface protein (MSP-3).

**Methods:**

These peptides were coupled to phosphatidylethanolamine (PE); the conjugates were intercalated into immunopotentiating reconstituted influenza virosomes (IRIVs) and then used for immunizations in mice to evaluate their capacity to elicit *P. falciparum *cross-reactive antibodies.

**Results:**

While all MSP-3-derived peptides were able to elicit parasite-binding antibodies, stabilization of turn structures by cyclization had no immune-enhancing effect. Therefore, further pre-clinical profiling was focused on FB-12, a PE conjugate of the linear peptide. Consistent with the immunological results obtained in mice, all FB-12 immunized rabbits tested seroconverted and consistently elicited antibodies that interacted with blood stage parasites. It was observed that a dose of 50 μg was superior to a dose of 10 μg and that influenza pre-existing immunity improved the immunogenicity of FB-12 in rabbits. FB-12 production was successfully up-scaled and the immunogenicity of a vaccine formulation, produced according to the rules of Good Manufacturing Practice (GMP), was tested in mice and rabbits. All animals tested developed parasite-binding antibodies. Comparison of ELISA and IFA titers as well as the characterization of a panel of anti-FB-12 monoclonal antibodies indicated that at least the majority of antibodies specific for the virosomally formulated synthetic peptide were parasite cross-reactive.

**Conclusion:**

These results reconfirm the suitability of IRIVs as a carrier/adjuvant system for the induction of strong humoral immune responses against a wide range of synthetic peptide antigens. The virosomal formulation of the FB-12 peptidomimetic is suitable for use in humans and represents a candidate component for a virosomal multi-valent malaria subunit vaccine.

## Background

The development of an anti-malarial vaccine represents one of the most important public health priorities. It is generally assumed that a multi-stage, multi-component vaccine is required to provide sufficient protection against *Plasmodium falciparum *malaria [1,2]. One approach is the design of a subunit vaccine that incorporates several synthetic peptide antigens for which there is evidence of protective immunity from in vitro parasite growth inhibition assays, experimental malaria infection models and/or immuno-epidemiological studies. Peptide-based vaccines could have many advantages compared to conventional vaccines, such as increased stability and safety and lower cost [3]. Ideally, synthetic subunit vaccines focus immune responses on antigenic determinants relevant for protection, thus avoiding the induction of deleterious immune responses as observed during *P. falciparum *infection [4]. However, the development of synthetic peptide vaccines is often hampered by limited intrinsic immunostimulatory properties and the lack of cross-reactivity of elicited antibodies with native target antigens.

Both problems can be addressed by developing synthetic peptide structures that induce cross-reactive antibodies against the parent malaria proteins and by coupling them to the surface of immunopotentiating reconstituted influenza virosomes (IRIVs) via a phosphatidylethanolamine (PE) anchor. IRIVs represent an innovative antigen delivery system derived from a mixture of natural and synthetic phospholipids and influenza surface glycoproteins. The suitability of IRIV as peptide carrier and adjuvant system for malaria peptidomimetics has been proven in several pre-clinical [4-10] and clinical [11,12] studies. Experience with two licensed vaccines based on virosomes has shown that IRIV based vaccines have an excellent safety profile and are highly immunogenic also in children and infants [13,14].

One of the target antigens for inclusion into a malaria vaccine is the *P. falciparum *merozoite surface protein (MSP-3). MSP-3 is a non-integral surface-associated protein that may be an important target for antibody-mediated protective immunity, as truncation of the MSP-3 gene reduces parasite invasion [15]. Antibodies to MSP-3 have shown parasite growth inhibitory activity in antibody dependent cellular inhibition (ADCI) assays and in a humanized SCID mouse *P. falciparum *infection model [16-20]. Cytophilic antibodies to polymorphic and conserved epitopes of MSP-3 were shown to be associated with reduced incidence of malaria in immuno-epidemiological studies [21-23]. MSP-3 vaccinated *Saimiri *and *Aotus *monkeys have been partially protected from lethal challenge with *P. falciparum *blood stage parasites [24,25].

This report describes the pre-clinical profiling of a virosomally-formulated synthetic peptidomimetics comprising amino acids 211-237 of *P. falciparum *MSP-3.

## Methods

### Animal studies

All procedures involving living animals were performed in accordance with the Rules and Regulations for the Protection of Animal Rights (Tierschutzverordnung) of the Swiss Bundesamt für Veterinärwesen.

### Mouse immunogenicity studies

Peptide synthesis and the preparation of peptide-loaded virosomes were done as described previously [7,9]. BALB/c mice were pre-immunized intramuscularly with inactivated influenza virus (1 μg HA per dose [A/Sing]). At least three weeks later they were immunized with peptide-loaded IRIVs (containing 5 μg PE-peptide) in intervals of at least two weeks. Blood was collected before each immunization and two weeks after the final injection.

### Rabbit immunogenicity studies

New Zealand rabbits were pre-immunized intramuscularly with inactivated influenza virus (10 μg HA per dose). Three weeks later they were immunized with peptide-loaded IRIVs (containing 10, 25 or 50 μg PE-peptide) in intervals of three weeks. Blood was collected before each immunization and three weeks after the final injection.

### Enzyme-linked immunosorbent assay (ELISA)

Polysorp™ microtiter plates (Nunc, Fisher Scientific, Wohlen, Switzerland) were coated overnight at 4°C with 100 μL of a 10 μg/mL solution of peptide-PE conjugate in PBS (pH 7.2). Wells were then blocked with 5% milk powder in PBS for 30 min at 37°C followed by three washings with PBS containing 0.05% Tween-20. Plates were then incubated with serial dilutions of anti-peptide mAbs, mouse or rabbit sera in PBS containing 0.05% Tween-20 and 0.5% milk powder for 2 h at 37°C. After washing, plates were incubated with alkaline phosphatase-conjugated goat anti-mouse IgG (γ-chain specific) antibodies (Sigma, St. Louis, MO) or with phosphatase-conjugated affinity-pure F(ab')_2 _fragment goat anti-rabbit IgG heavy-chain antibodies (KPL, Guildford, UK) for 1 h at 37°C. Phosphatase substrate (1 mg/mL p-nitrophenyl phosphate (Sigma)) in buffer (0.14% Na_2_CO_3_, 0.3% NaHCO_3_, 0.02% MgCl_2_, pH 9.6) was added and incubated at room temperature. The optical density (OD) of the reaction product was recorded after appropriate time at 405 nm using a microplate reader (Sunrise™, Tecan Trading AG, Switzerland).

### NH4SCN elution ELISA

Avidity ELISA analyses with peptide-PE conjugates were performed essentially as described before [26]. After coating and blocking, rabbit serum samples were added in triplicates at constant dilutions (approx. halfmax titer). After a wash step, plates were incubated 15 min with NH_4_SCN diluted in 0.1 M NaH_2_PO_4 _buffer (pH 6) at the following molarities: 5 M, 4 M, 3 M, 2 M, 1 M, 0.5 M, 0.25 M. Control wells were incubated with 0.1 M NaH_2_PO_4 _buffer without NH_4_SCN. After washing, plates were incubated with alkaline phosphatase-conjugated affinity-pure F(ab')_2 _fragment goat anti-rabbit IgG heavy-chain antibodies (KPL, Guildford, UK) and developed with phosphatase substrate solution. The avidity index corresponds to the NH_4_SCN concentration (M) eluting 50% of the bound antibodies.

### Generation of anti-FB-12 monoclonal antibodies (mAbs)

Three days before cell fusion, a BALB/c mouse immunized with FB-12-PE loaded IRIV received an intravenous booster injection. From the sacrificed mouse the spleen was aseptically removed and a spleen cell suspension in IMDM was mixed with PAI mouse myeloma cells as a fusion partner. Spleen and myeloma cells in a ratio of 1:1 were centrifuged; having the supernatant discarded, the pellet was mixed with 1 mL pre-warmed polyethylene glycol 1500 sterile solution. After 60 s 10 mL of culture medium were added. After 10 min cells were suspended in IMDM containing hypoxanthine, aminopterin, thymidine, and 20% foetal bovine serum and cultured in 96-well tissue culture plates. Cells secreting FB-12-specific IgG were identified by ELISA coated with FB-12-PE conjugate. From two independent fusions five hybridoma cell lines producing peptide specific mAbs were identified and cloned twice by limiting dilution. MAbs were purified from spent culture supernatant of the hybridoma clones by protein A affinity chromatography (HiTrap rProtein A FF, Amersham Biosciences). Purified mAbs were dialyzed against PBS, aliquoted, and stored at -80°C.

### Indirect immunofluorescence assay (IFA)

In vitro cultivated *P. falciparum *parasites were washed and mixed with two volumes of a solution containing 4% paraformaldehyde and 0.1% Triton X-100. Droplets of 40 μL of cell suspension were added to each well of a diagnostic microscope slide (Flow Laboratories, Baar, Switzerland) and incubated for 30 min at room temperature. Cells were blocked with blocking solution containing 100 mg/mL fatty acid-free bovine serum albumin in PBS. Immunostaining was performed by incubating the wells with 25 μL of an appropriate mAb or serum dilution in blocking solution in a humid chamber for one hour at room temperature. After five washes with blocking solution, 25 μL of 5 μg/mL indocarbocyanine dye-conjugated affinity-pure F(ab')_2 _fragment goat anti-mouse IgG heavy-chain antibodies (Jackson ImmunoResearch Laboratories, West Grove, Pa.), diluted in blocking solution were added to the wells and incubated for one hour at room temperature. Finally, the wells were washed five times, mounted with ProLong^® ^Gold antifade reagent with DAPI (Invitrogen) and covered with a coverslip. Antibody binding and DNA staining were assessed by fluorescence microscopy.

### Sodium dodecyl sulfate-polyacrylamide gel electrophoresis (SDS-PAGE) and immunoblotting

Parasite lysates were prepared by saponin lysis of *P. falciparum *infected erythrocytes. Cultured parasites were washed with RPMI medium. Pelleted infected red blood cells were lysed by mixing with a large volume (adjusted to 5% haematocrit) of 0.015% (wt/vol) saponin in PBS and incubated on ice for 20 min. Finally, the pelleted parasites were resuspended in PBS and stored at -80°C until further use.

A total of 50 μL of parasite lysate was solubilized in an equal volume of 2× loading buffer (1.7 mL of 0.5 M Tris-HCl [pH 6.8], 2 mL of glycerol, 4.5 mL of 10% sodium dodecyl sulfate, 1 mL of β-mercaptoethanol, 0.8 mL of bromophenol blue [0.3%, wt/vol]) and heated to 95°C for 10 min. Proteins were separated on an SDS-PAGE minigel and electrophoretically transferred to a nitrocellulose filter by semidry blotting. Blots were blocked with PBS containing 5% milk powder and 0.1% Tween 20 overnight at 4°C. The filter was cut into strips and incubated with appropriate dilutions of mAbs or immune serum in blocking buffer for 2 h at room temperature. After several washing steps, filter strips were incubated with goat anti-mouse IgG horseradish peroxidase conjugated Ig (Bio-Rad Laboratories, Hercules, CA) or horseradish peroxidase conjugated goat anti-rabbit IgG heavy and light chain antibodies (Bio-Rad Laboratories, Hercules, CA) for 1 h. Blots were developed using the ECL system according to manufacturer's instructions.

### *In vitro *growth inhibition assays

*In vitro *growth inhibition assays with *P. falciparum *strain K1 were conducted essentially as described [7]. Briefly, synchronous late trophozoites were diluted with fresh red blood cells to result in 0.5% parasitaemia and mixed with purified mAb. The final haematocrit in cultures was adjusted to 0.5%. Each culture was set up in sextuplicate in 96-well flat-bottomed culture plates. After 96 h, the plates were centrifuged at 180 × g for 5 min, and the culture supernatants were discarded. Pelleted erythrocytes were resuspended in 200 μL of PBS supplemented with 15 μg of hydroethidine fluorescent vital stain (Polysciences Inc., Warrington, Pa.) per mL and incubated at 37°C for 30 min. The erythrocytes were washed with PBS and analysed in a FACSscan flow cytometer (Becton Dickinson, San Jose, Calif.) with CellQuest 3.2.1 fl software. A total of 30,000 cells per sample were analysed. Percent inhibition was calculated from the geometric mean parasitaemia of sextuplicate test and control wells as 100 × [(control - test)/control].

## Results

### Design and synthesis of peptide-PE conjugates

**Figure 1:**
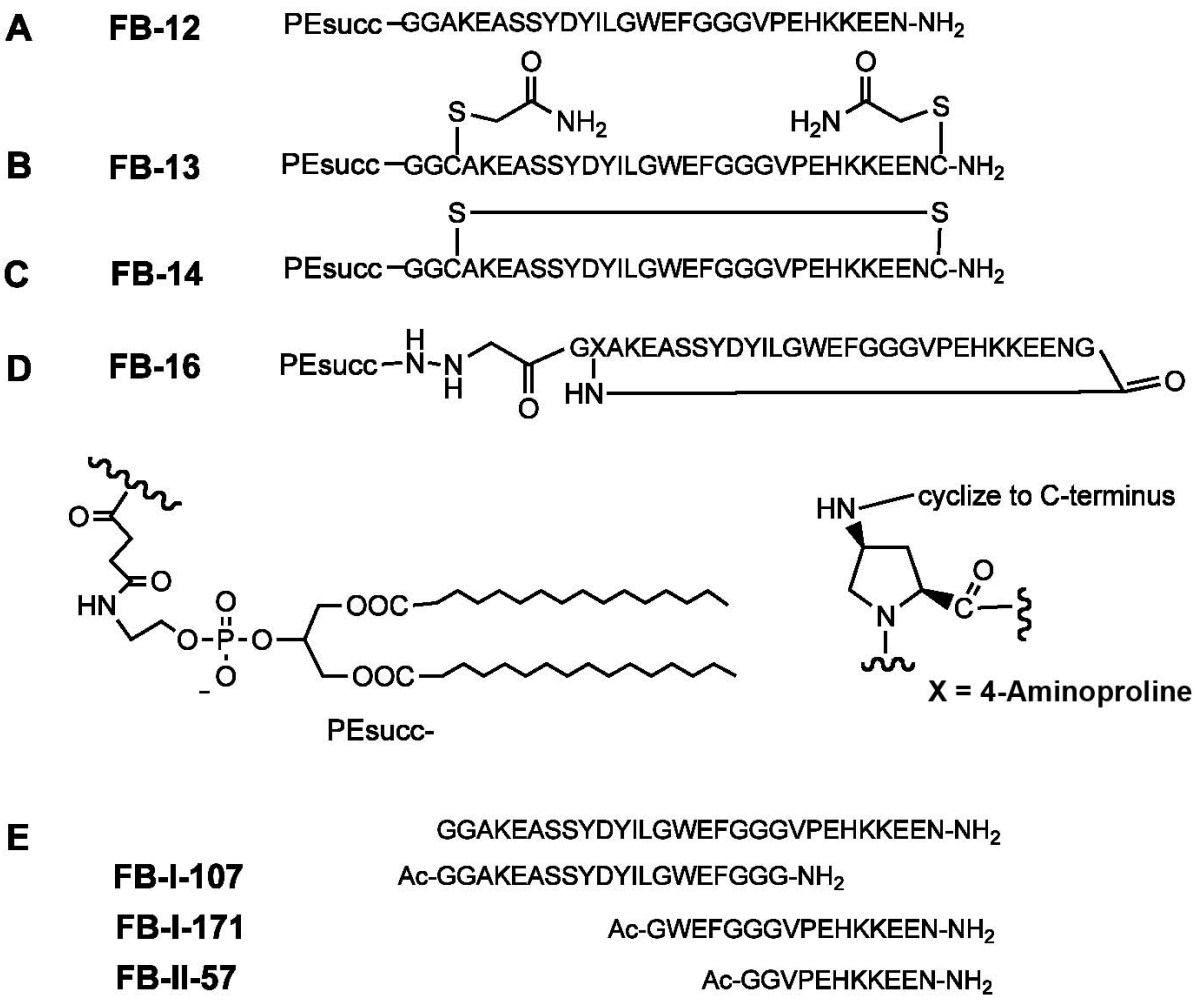
**Structures of the synthetic peptides and peptide-PE conjugates prepared in this study**.

A series of phosphatidylethanolamine (PE)-peptide conjugates comprising amino acids 211-237 of the *P. falciparum *vaccine candidate antigen MSP-3 was designed and synthesized, since previous studies [27-29] indicated that parasite inhibitory epitopes exists between the conserved residues 212-257. These peptides were linked via two N-terminally added glycine residues to PE, incorporated into IRIV's membrane, presenting the antigen on the surface, and then used for immunizations. Figure 1 gives an overview over all MSP-3 derived peptides and peptide-PE conjugates that have been used for pre-clinical profiling in this study.

In addition to FB-12, a PE conjugate of MSP-3_211-237 _of *P. falciparum *strain K1 (Figure 1A), identical peptide sequences flanked by two additional cysteine residues were synthesized. These conjugates were designated FB-13 (Figure 1B) with both cysteine residues being alkylated with iodoacetamide and FB-14 (Figure 1C) with both cysteine residues linked to each other by an internal disulfide bond. FB-13 was expected to have comparable immunological properties as FB-12 and was produced as a control for the evaluation of the effects of cyclization. Finally, an alternative cyclization strategy was developed in order to achieve a cysteine-independent cyclization of MSP-3_211-237_. The resulting peptidomimetic, designated FB-16, was cyclized to one large loop by introducing (2S, 4S)-4-aminoproline and linking the peptide C-terminus to the 4-amino group (Figure 1D).

### Immunogenicity of virosomal formulations of MSP-3-derived synthetic peptide-PE conjugates in mice

**Figure 2:**
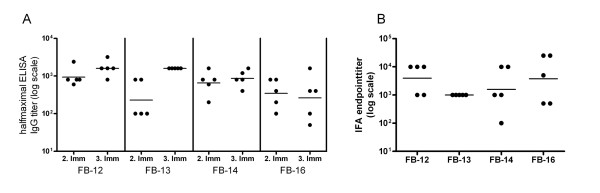
**Immune responses elicited by virosomal formulations of MSP-3_211-237 _derived peptide-PE conjugates**. Development of anti-peptide-PE IgG responses in peptide ELISA (A). Sown are halfmaximal IgG titers for individual animals and the geometric mean (line) after the second and third vaccination. Cross-reactivity of anti-peptide-PE IgG responses with cultured *P. falciparum *blood-stage parasites (B). Shown are individual IFA endpoint titers after the third immunization, lines representing the geometric mean. None of the pre-immune sera was positive in ELISA or IFA.

BALB/c mice were immunized three times with virosomally formulated peptide-PE conjugates. Pre-immune sera and sera taken after the second and third immunization were analysed for the development of peptide specific IgG in ELISA and for parasite cross-reactive IgG in IFA with in vitro cultivated blood stage parasites. Already after two immunizations all mice immunized with either FB-12, FB-13, FB-14 or FB-16 produced antibodies that showed specific reactivity with the respective immunogen in ELISA (Figure 2A). Moreover, all sera were consistently cross-reactive with schizonts (Figure 2B). For all peptides, mean serum antibody titers for the respective immunogen in ELISA were enhanced by the third immunization. Since structural stabilization by cyclization of the peptide did not enhance immunogenicity, further preclinical development was focused on the linear peptide FB-12.

### Generation and characterization of anti-FB-12 mAbs

For a structural characterization of anti-FB-12 antibody responses, a panel of anti-FB-12 mAbs was generated. Five hybridoma clones producing mAbs (designated DV2.2 and DV3.1 to DV3.4) were selected using a FB-12 specific peptide ELISA. DV2.2 and the DV3 series of mAbs were obtained from independent fusions with spleen cells of two FB-12 immunized BALB/c mice. All five mAbs bound to all linear and cyclic peptides that comprised aa 211-237 of MSP-3 (Table 1). In order to map the epitopes of the anti-FB-12 mAbs in more detail, reactivity with truncated fragments of FB-12 (Figure 2E) was analysed. While loss of the C-terminal portion of the MSP-3_211-237 _sequence in peptide aa 211-228 (structure FB-I-107) did not affect mAb binding, none of the mAbs reacted in ELISA with a N-terminally truncated peptide comprising aa 227-237 (structure FB-II-57).

**Table 1:** Cross-reactivity of anti FB-12 mAbs with FB-12-derived peptides in ELISA and with *P. falciparum *blood-stage parasites in IFA and Western blotting (WB) analysis.

**mAb**	**full length peptides**				**truncated peptides**			**Schizont**	
	**FB-12**	**FB-13**	**FB-14**	**FB-16**	**FB-II-57**	**FB-I-171**	**FB-I-107**		
	**211-237**	**211-237**	**211-237**	**211-237**	**227-237**	**222-237**	**211-228**	**IFA**	**WB**
	
**DV2.2 **(IgG_1_/k)	+	+	+	+	**-**	**-**	**+**	+	+
	
**DV3.1 **(IgG_1_/l)	+	+	+	+	**-**	**+**	**+**	+	+
	
**DV3.2 **(IgG_1_/l)	+	+	+	+	**-**	**-**	**+**	+	+
	
**DV3.3 **(IgG_1_/l)	+	+	+	+	**-**	**+**	**+**	+	-
	
**DV3.4 **(IgG_2b_/l)	+	+	+	+	**-**	**+**	**+**	+	+

Differential binding of mAbs was observed when testing for reactivity with a N-terminally less truncated peptide comprising aa 222-237 (FB-I-171). While mAbs DV3.1, DV3.3 and DV3.4 showed reactivity with this peptide, mAbs DV2.2 and DV3.2 were still negative. All five anti-FB-12 mAbs cross-reacted with parasite expressed MSP-3, as demonstrated by IFA. With the exception of mAb DV3.3, all mAbs were also reactive with the MSP-3 protein in Western blot analysis with total lysates of *P. falciparum *blood stage parasites (Table 1).

**Figure 3:**
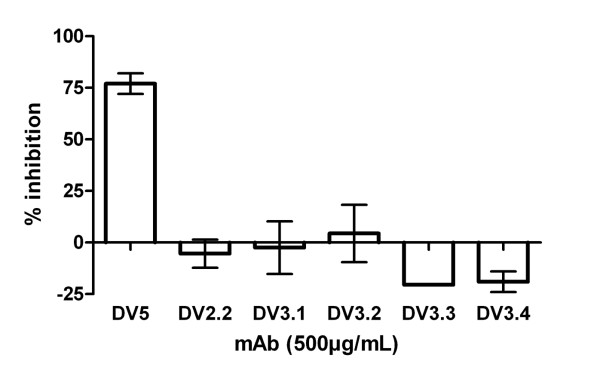
***In vitro *growth inhibition assay**. Blood stage parasites were synchronized and cultured in sextuplicate in the presence of mAbs or equal volumes of PBS as control. Numbers of parasites were determined by FACS after two blood stage cycles and inhibition calculated relative to the PBS control. Shown are the means ± S.D. of two experiments. The growth-inhibitory mAb DV5, specific for apical membrane antigen 1, was used a positive control [7].

*In vitro *growth inhibition assays with blood stage parasites were performed to assess a parasite inhibitory effect of the anti-FB-12 mAbs. In contrast to the growth-inhibitory mAb DV5 specific for apical membrane antigen 1 [7], no growth inhibition was found in this test system with any of the anti-FB-12 mAbs tested (Figure 3). Previous reports have shown that anti-MSP-3 antibodies have no direct effect upon merozoite invasion but can cooperate with blood monocytes to inhibit *P. falciparum in vitro *growth [16,18].

### Immunogenicity of peptide FB-12 in rabbits

**Figure 4:**
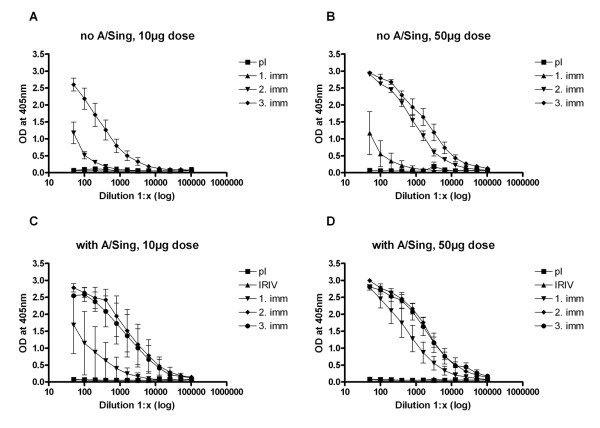
**Development of anti-peptide IgG responses in ELISA after immunization with IRIV-formulated FB-12 in rabbits**. Groups of three animals were immunized with two different doses (10 μg [A, C], and 50 μg [B, D] peptide-PE conjugate) without (A, B) or with (C, D) pre-immunization with 10 μg inactivated influenza virus (A/Sing). Shown are serial dilutions (means +/-SD) of rabbit sera taken two weeks after first, second and third immunization. No peptide-specific IgG responses were found in pre-immune sera.

New Zealand rabbits were immunized with two different doses of virosomally formulated FB-12 (10 μg, and 50 μg of peptide-PE conjugate) with or without pre-immunization with 10 μg inactivated influenza virus (A/Sing). Consistent with the results obtained from the mouse immunization experiments all FB-12 immunized rabbits developed IgG antibodies that interacted both with the peptide immunogen in ELISA (Figure 4) and blood stage parasites in IFA. In particular in animals not pre-immunized with A/Sing the dose of 50 μg was superior to the 10 μg dose (Figure 4A and Figure 4B). In contrast to the 10 μg group (Figure 4A), rabbits receiving the 50 μg dose developed an anti-FB-12 IgG response already after one immunization (Figure 4B). Booster effects after the second and third immunization were observed both in the low and high dose groups with variation in the maximum antibody titers. Pre-immunization with A/Sing (Figure 4C and Figure 4D) improved the immunogenicity of FB-12 in rabbits especially for the low dose group. In these primed rabbits both vaccine doses induced an anti-peptide IgG response already after the first immunization but with higher titers in the 50 μg group. Comparable anti-FB-12 IgG titers were reached after the second immunization in both A/Sing primed dose-groups and no significant increase in titers resulted from the third immunization.

### Immunogenicity of a virosomal formulation of a FB-12 produced according to the rules of GMP

**Figure 5:**
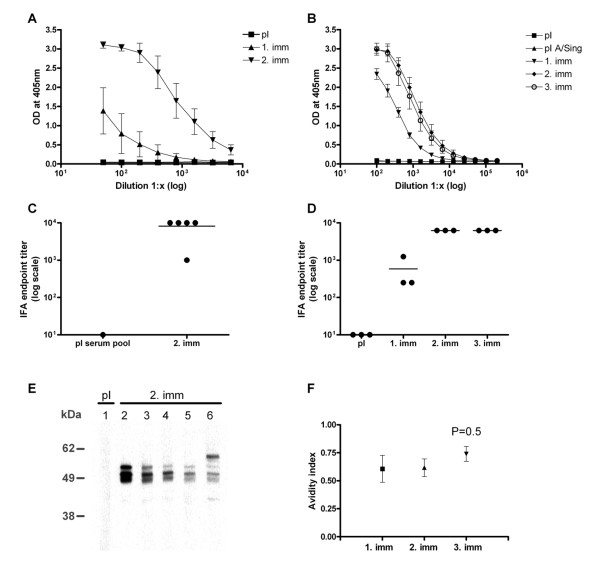
**Immunogenicity of a virosomal FB-12 formulation produced according to the rules of GMP**. Development of anti-peptide IgG responses in mice (A) and in rabbits (B). Shown are serial dilutions of sera in peptide-ELISA (means +/-SD). Cross-reactivity of anti-FB-12 IgG responses of mice (C) and rabbits (D) with cultured *P. falciparum *blood-stage parasites in IFA. E: Western-blot analysis of mouse serum samples with blood stage parasite lysate. Pooled pre-immune serum (E, lane 1) and individual serum samples (E, lanes 2-6) collected two weeks after the second immunization were used at a dilution of 1:10'000. F: Mean avidity indices for anti-FB-12 IgG responses of rabbits three weeks after the first, second and third immunization. The avidity index corresponds to the NH_4_SCN concentration (M) where 50% of the bound antibodies are eluted. Shown are means +/-SD. Wilcoxon signed-rank test was used to calculate the statistical significance of a difference in avidity between the third and the first vaccination.

**Figure 6:**
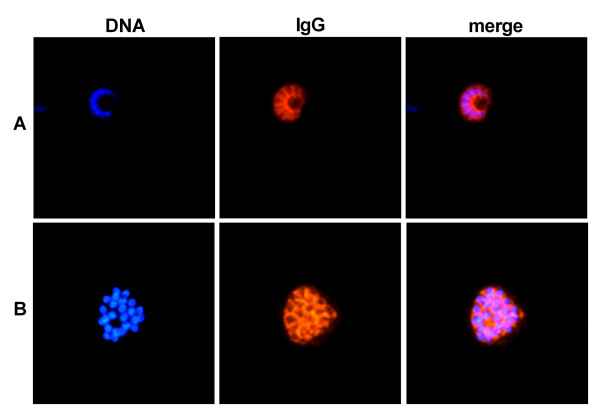
**Development of MSP-3 cross-reactive IgG in IFA with *P. falciparum *schizonts upon immunization with FB-12-PE loaded IRIV produced according to the rules of GMP**. Immune sera of mice (A) and rabbits (B) taken three weeks after the second immunization were tested for parasite binding. Sera were diluted 1:1000 and representative examples are shown. The left panel shows DNA staining with DAPI (blue), the middle panel Cy3-immunofluorescence staining (red), the right picture is the merge.

As a next step, synthesis of FB-12 was up-scaled and the immunogenicity of a batch produced according to the rules of GMP was tested. A/Sing primed BALB/c mice were immunized twice with 5 μg of virosomally formulated FB-12. Already one immunization elicited detectable titers of anti-FB-12 IgG in peptide ELISA and a second immunization led to a strong titer increase (Figure 5A). All FB-12 immunized mice developed high IFA IgG antibody titers (Figure 5C) and the observed fluorescence staining pattern was typical for a merozoite surface staining (Figure 6A). All anti-FB-12 immune sera were consistently cross-reactive with MSP-3 expressed by blood stage schizonts as detected in Western blot analysis with parasite lysate (Figure 5E). The observed triple band staining pattern is characteristic for the processed MSP-3 protein [30].

Pre-clinical profiling was continued by immunogenicity studies in a second species, namely rabbits. Already one immunization of A/Sing primed New Zealand rabbits with 25 μg of FB-12-loaded IRIV elicited high titers of anti-FB-12 IgG in ELISA (Figure 5B) and schizont cross-reactive IgG in IFA (Figure 5D and Figure 6B). While a second immunization led to a strong ELISA and IFA titer increase, a third immunization had no further booster effect. The mean avidity index, a measure for antibody affinity maturation, of the FB-12-induced IgG response in rabbits increased slightly after the third immunization (Figure 5F).

## Discussion

Previously, an iterative optimization process was used to develop synthetic peptides mimicking the native structure of surface loops of leading *P. falciparum *vaccine candidate antigens [7-10,31]. Furthermore, a technology was developed for the delivery of PE-coupled synthetic peptide antigens on the surface of influenza virosomes (IRIVs). A phase I clinical trial demonstrated safety and parasite cross-reactive immunogenicity of two IRIV-formulated peptides derived from the circumsporozoite surface protein (CSP) and the apical membrane antigen 1 (AMA1) [11]. Purified immunoglobulins from CSP immunized volunteers inhibited substantially sporozoite migration and invasion of hepatocytes in vitro [4]. Combined delivery of the two virosomal constructs did not interfere with immunogenicity of either peptide, demonstrating the suitability of the IRIV system for development of multi-valent subunit vaccines. In a phase IIa trial the two combined IRIV-formulated peptides showed evidence of vaccine-induced blood-stage efficacy for the first time in a sporozoite challenge study [12]. The malaria naive study participants were not completely protected from malaria, but lower rates of parasite growth and the presence of morphologically abnormal parasites (crisis forms) were significantly observed in vaccinated volunteers. While these clinical trials support the concept of using peptide-loaded IRIVs for vaccination in humans, it is assumed that additional key *P. falciparum *antigens need to be added to produce an effective multi-valent malaria vaccine. In the case of MSP-3, choice of the sequence stretch incorporated was guided by structural considerations and pre-existing knowledge on epitopes of parasite inhibitory antibodies [18,19,27-29].

In this study, immunogenicity of a small library of linear and constrained MSP-3-derived peptides was compared. In particular, it was investigated, whether stabilization of turn structures by cyclization improves the ability of the peptides to elicit antibodies that cross-react with the native target protein. Suitability of the linear peptide indicates that intramolecular interactions lead to a correct folding of the linear FB-12 peptide and that anchoring to the surface of IRIVs has no deleterious effects on this process. In contrast, delivery of peptide antigens adsorbed to alum or other adjuvants, may disturb the structure even of conformationally restricted peptides [6].

Since FB-12 was also the easiest structure to synthesize, further development was focused on this peptide-PE conjugate as a new candidate for incorporation into a virosomal multi-valent subunit malaria vaccine. Virosomally formulated FB-12 elicited high titers of blood stage parasite cross-reactive antibodies both in all inbred mice and in all rabbits with diverse immunogenetic backgrounds tested.

In order to test whether a pre-existing immunity against the virosomal carrier system influences its efficacy, animals were immunized with influenza antigens prior to immunization with the virosomal malaria vaccine candidate antigens. These experiments have shown that influenza pre-immunity enhances immune responses against the malaria antigen. Possible explanations are (i) opsonization of IRIVs with pre-existing anti-influenza antibodies, leading to enhanced uptake by antigen presenting cells and (ii) activation of influenza-specific memory T cells, providing T cell help to FB-12 specific B cells. The effect was most pronounced after the first immunization and leveled of afterwards, presumably because anti-influenza immune responses are induced by the first immunization with antigen-loaded virosomes. These results show that pre-immunity to the virosomal antigen delivery system enhances the immune response but is not a pre-requisite.

Influenza specific immune responses have been found in African and Asian children [32-34] and experience with two licensed vaccines based on virosomes have shown that the technology is suitable for use in children and infants [13,14].

Production of sets of mAbs against a peptide antigen is instrumental for evaluating the quality of vaccine candidates [6,10]. One key criterion is the proportion of parasite binding mAbs within the population of peptide reactive antibodies elicited. In the case of FB-12 all five generated anti-FB-12 mAbs derived from two mice bound to blood stage parasites. Evidence for diversity of the fine specificity of the mAbs came from the analysis of their cross-reactivity with partial fragments of FB-12. Results indicate that for all mAbs the most essential amino acids for recognition are covered by the N-terminal portion of FB-12 (aa 211-228) and that mAbs DV3.1, DV3.3 and DV3.4 seem to interact primarily with aa 222-228.

Analyses of the IgG subclass profiles of the induced FB-12-specific mAbs showed a predominance of the IgG_1 _isotype. Despite binding to the blood stage parasites, the anti-FB-12 mAbs had no effect on parasite replication *in vitro*. It has been reported previously that anti-MSP-3 inhibitory effects involve monocytes in a process called antibody-dependent cellular inhibition (ADCI) [16,18]. It would therefore be interesting to test our mAbs in an ADCI test system. However, the correlation of *in vitro *growth inhibitory activities of antibodies with their potential protective capacity *in vivo *is incompletely understood.

Production of FB-12 was successfully up-scaled and a formulation produced according to the rules of GMP was immunogenic, leading to seroconversion in all immunized mice and rabbits. IFA titers in mice and rabbits closely followed ELISA titers, indicating a close relationship between FB-12 and the natural conformation of MSP-3. In an attempt to gain evidence for affinity maturation of FB-12 specific B cells upon repeated immunization of rabbits with FB-12, binding inhibition ELISAs with chaotropic salt were performed to measure avidity of peptide-specific IgG. Affinity maturation, indicative for memory B cell formation, was evidenced by a slight increase in anti-FB-12 antibody avidity over the course of immunization. In contrast to mice and humans, in rabbits immunoglobulin genes diversify by both somatic hypermutation and somatic gene conversion during the course of a T cell-dependent immune response [35].

## Conclusion

Taken together, it was possible to induce parasite cross-reactive antibodies with a human compatible formulation of a synthetic MSP-3-derived peptide in mice and rabbits. Clinical grade material has been produced and is available for phase I clinical testing.

## Competing interests

The authors declare that they have no competing interests.

## Authors' contributions

JAB, RZ and GP designed the research. MT, MSM, SAS, NW, DV and FB performed the research. MT, MSM, SAS, NW, JAB, RZ and GP analysed the data. MT and GP wrote the paper with contributions from the other authors. All authors read and approved the final manuscript.
